# The Potential Impact of SGLT2-I in Diabetic Foot Prevention: Promising Pathophysiologic Implications, State of the Art, and Future Perspectives—A Narrative Review

**DOI:** 10.3390/medicina60111796

**Published:** 2024-11-01

**Authors:** Giuseppe Miceli, Maria Grazia Basso, Andrea Roberta Pennacchio, Elena Cocciola, Chiara Pintus, Mariagiovanna Cuffaro, Martina Profita, Giuliana Rizzo, Mariachiara Sferruzza, Antonino Tuttolomondo

**Affiliations:** 1 Department of Health Promotion, Mother and Child Care, Internal Medicine and Medical Specialties (ProMISE), Università degli Studi di Palermo, Piazza delle Cliniche 2, 90127 Palermo, Italy; mariagbasso.92@gmail.com (M.G.B.); pennacchio.andrea@libero.it (A.R.P.); elena.cocciola@gmail.com (E.C.); chiarapintus1809@gmail.com (C.P.); marinecuffaro@libero.it (M.C.); martinaprofita9@gmail.com (M.P.); giulianarizzo992@gmail.com (G.R.); maki.sferruzza@gmail.com (M.S.); bruno.tuttolomondo@unipa.it (A.T.); 2 Internal Medicine and Stroke Care Ward, University Hospital, Policlinico “P. Giaccone”, 90127 Palermo, Italy

**Keywords:** SGLT2-i, diabetic foot, neuropathy, atherosclerosis, PAD

## Abstract

The impact of diabetic foot (DF) on the healthcare system represents a major public health problem, leading to a considerable clinical and economic burden. The factors contributing to DF’s development and progression are strongly interconnected, including metabolic causes, neuropathy, arteriopathy, and inflammatory changes. Sodium–glucose cotransporter 2 inhibitors (SGLT2-i), novel oral hypoglycemic drugs used as an adjunct to standard treatment, have recently changed the pharmacological management of diabetes. Nevertheless, data about the risk of limb amputation, discordant and limited to canagliflozin, which is currently avoided in the case of peripheral artery disease, have potentially discouraged the design of specific studies targeting DF. There is good evidence for the single immunomodulatory, neuroprotective, and beneficial vascular effects of SGLT2-i. Still, there is no clinical evidence about the early use of SGLT2-i in diabetic foot due to the lack of longitudinal and prospective studies proving the effect of these drugs without confounders. This narrative review aims to discuss the main evidence about the impact of SGLT2-i on the three complications of diabetes implicated in the development of DF, the state of the art, and the potential future implications.

## 1. Introduction

Diabetic foot (DF) is a chronic complication of diabetes that consists of an anatomic–functional impairment of the foot related to alterations in peripheral blood vessels and nerves in the lower limbs. Sensory and motor neuropathy, trauma, and peripheral artery disease ultimately lead to the development of foot ulceration (neuropathic and ischemic) and Charcot neuroarthropathy [1].

Worldwide, a third of people with diabetes develop a diabetic foot ulcer in the course of their lifetime with a relapse rate at one year of 40% [2]. The impact of this complication is underscored by the high prevalence of infections with subsequent amputations and mortality: 85% of lower-limb amputation procedures in people with diabetes are preceded by a foot ulcer [3] with a 5-year mortality of 56.6% for patients undergoing major amputation [2]. From this perspective, the impact of diabetic foot on the healthcare system represents a major public health problem, leading to a considerable clinical and economic burden.

In patients with type 2 diabetes mellitus (T2DM), lifestyle changes and oral hypoglycemic agents, of which metformin represents the first-line therapy, demonstrate glycemic control and contribute to determining reduction in terms of complications and hospitalization.

Sodium–glucose cotransporter 2 inhibitors (SGLT2-i), novel oral hypoglycemic drugs used as an adjunct to standard treatment, have recently changed the pharmacological management of diabetes. They have become a critical component of diabetes therapy due to their unique mechanism of action and the wide range of benefits including lowering blood glucose levels independent of insulin action, reducing cardiovascular events and the risk of hospitalization for heart failure, slowing the progression of diabetic kidney disease by reducing albuminuria, and improving the overall quality of life for patients by addressing both metabolic and cardiovascular risks [4]. They promote glucose excretion by reducing glucose absorption at the proximal renal tubule level, reducing glycated hemoglobin values, and weight loss [5]. In addition to the hypoglycemic effect, several studies have shown that SGLT2-i have a pleiotropic outcome in preventing hospitalization and death in T2DM patients through the nephroprotective and cardiovascular protective role and anti-inflammatory effects but with some alerts connected to amputation risk [6] whose importance has been outlined and discussed in detail in some reviews [7,8]. Since the complex pathophysiology of DF encompasses atherosclerotic processes, neuropathy, and infection involving well-known inflammatory and thrombotic pathogenetic events, it is to date only potentially speculative to hypothesize their effectiveness also in the clinical setting of diabetic foot syndrome (DFS). Nevertheless, it could be highly suggestive of a parallelism between the peripheral vascular effects of SGLT2-i on clinical outcomes related to DFS, such as reduction in amputation rate, rehospitalization, and infection, and also of the impacts on cardiovascular morbidity that have underlined the role of DFS as a cardiovascular risk indicator in diabetics [9]. This review aims to discuss the main evidence about the effects of SGLT2-i on the three complications of T2DM implicated in the development of DF and the potential future implications. After a brief presentation of both the pathophysiology of DF and the pharmacology of this promising anti-diabetic oral agent, we will subsequently discuss the effect of SGLT2-i on the multifaceted components of diabetic foot, such as the immunomodulatory effect on the vascular system, neuropathy, peripheral artery disease, risk of infection, and risk of amputation. Finally, we critically appraise the most recent evidence in clinical practice and discuss the potential knowledge gap that must be addressed in future investigations.

## 2. Methods

The study collection for the review composition was conducted through research on Pubmed and Google Scholar. Only manuscripts written in English from January 2010 to July 2024 were considered. Keywords used for the research were as follows: “SGLT2-inhibitor and neuropathy”, “SGLT2-inhibitor and atherosclerosis”, “SGLT2-Inhibitor and immunomodulation”, “SGLT2-inhibitor and amputation”, SGLT”-inhibitor and infection” SGLT2-inhibitors and peripheral artery disease”, SGLT2-inhibitors and diabetic foot”. All kinds of works, such as clinical and experimental studies, reviews, systematic reviews, and metanalysis, were considered. No case reports or case series were considered. Artificial intelligence was not used for study selection. This narrative review is organized into distinct chapters that provide an overview of the mechanisms behind the development of diabetic foot in individuals with diabetes mellitus, highlighting the roles of oxidative stress and hyperglycemia. It discusses the effects of SGLT2 inhibitors on endothelial cells, inflammasomes that trigger inflammation in both humans and animals, and the balance between cell death and survival. Additionally, it explores the involvement of specific signaling molecules in these processes, alongside the general mechanisms of action of SGLT2 inhibitors and their impact on atherosclerosis. The review also addresses how SGLT2 inhibitors influence immunocompetent cells, which play a key role in the disease’s pathogenesis and symptoms, as well as their effects on cellular aging mechanisms. Furthermore, it details the processes by which these inhibitors affect neuropathy and angiopathy, the two main clinical manifestations of diabetes in the extremities, and highlights the risks associated with their use in individuals with diabetes.

## 3. Pathophysiology of Diabetic Foot

The factors contributing to DF’s development and progression are strongly interconnected, including metabolic causes, neuropathy, arteriopathy, and inflammatory changes. Some processes cause peripheral nerve damage: adenosine triphosphate (ATP) deficiency, protein kinase C (PKC) activity, the polyol pathway, and oxidative stress. Inadequate ATP levels hamper axonal transport, particularly in mitochondria-rich axons that provide nerve energy, and they cannot counter excessive oxidative stress. As a consequence, there is axonal degeneration or apoptosis [10].

Hyperglycemia activates PKC [11], promoting diabetic microvascular complications and overactivity of matrix metalloproteinases, which create a disorganized extracellular matrix, insufficient to support wound healing [12].

At the same time, the hyperglycemic state determines an increase in the action of the enzymes aldose reductase and sorbitol dehydrogenase, and it results in the conversion of intracellular glucose to sugar products: sorbitol and fructose. Sorbitol excess reduces Na^+^K^+^-ATPase activity, thereby diminishing nerve cell reserves; accumulated fructose accelerates nicotinamide adenine dinucleotide phosphate consumption, essential for glutathione generation [13]. This alteration results in decreased antioxidant levels and increased reactive oxygen species (ROS) production. In the context of hyperglycemia, elevated levels of ROS, notably superoxide radicals, are also determined by inhibiting the enzyme NO synthase, leading to the reduction in endothelial nitric oxide (NO) [14,15,16]. Inflammation plays a crucial role in the pathogenesis of diabetic foot complications, largely through its effects on endothelial function and adipo-inflammatory dysregulation [16]. In diabetes, chronic high blood glucose levels trigger systemic inflammation, which impairs endothelial function by disrupting the balance of nitric oxide and promoting oxidative stress. This endothelial dysfunction compromises vascular health, reducing blood flow and impairing wound healing [17]. Concurrently, adipo-inflammatory dysfunction—characterized by the release of proinflammatory cytokines from adipose tissue—further exacerbates this process. The resulting inflammatory milieu not only impairs tissue repair but also fosters an environment conducive to infection and ulcer formation, thus significantly contributing to the morbidity associated with diabetic foot. Immune cells, including neutrophils and macrophages, contribute to the prolonged inflammatory phase in DF ulcers (DFUs) through the release of neutrophil extracellular traps and abnormal macrophage polarization, both of which hinder wound healing. Additionally, DFUs are more prone to biofilm formation, which further disrupts the healing process by triggering chronic inflammation, impairing macrophage phagocytosis, inhibiting keratinocyte proliferation and migration, and promoting the transfer of antimicrobial resistance genes [18]. The combined action of NO and superoxide results in the production of peroxynitrite, which, mediating lipid peroxidation, leads to heightened concentrations of low-density lipoproteins, the development of microcirculation atherosclerosis (ATS), elevated inflammation, abnormal intimal growth, platelet aggregation, and thrombosis [19]. Furthermore, the formation of advanced glycation end products is now recognized as a key pathophysiological mechanism in the development of diabetic complications [20]. Several pathways have been proposed to explain how advanced glycation end products contribute to these complications. These include the accumulation of advanced glycation end products in the extracellular matrix, leading to abnormal cross-linking; the binding of circulating, advanced glycation end products to receptors on various cell types, which activates crucial cell signaling pathways and alters gene expression; and intracellular advanced glycation end product formation, which reduces nitric oxide availability and impairs the function of growth factors. Over the past decade, experimental studies [21] have demonstrated that advanced glycation end product formation contributes to the pathogenesis of both microvascular and macrovascular complications, diabetic neuropathy, and impaired wound healing. Moreover, recent clinical studies [22,23,24] have highlighted glycation as a significant factor in developing complications that increase the risk of DFUs. Not surprisingly, a recent study revealed that patients with DF exhibit proinflammatory and prooxidative conditions, evidenced by elevated levels of inflammatory biomarkers, an increased neutrophil-to-lymphocyte ratio, higher serum prooxidants, and a simultaneous reduction in antioxidative mechanisms. The findings also indicated that a heightened prooxidant–antioxidant balance and neutrophil-to-lymphocyte ratio are linked to the presence of DF [25,26].

These factors predispose to the onset of ischemia, which increases the risk of skin ulcers. Insufficient peripheral blood supply and elevated ROS levels delay wound healing and exacerbate infections [27]. Among microorganisms that can trigger DF infections, the most prevalent are *Staphylococcus aureus* and methicillin-resistant *S. aureus* (MRSA), found in 16.78% to 30% of cases [28]. Although MRSA infections do not appear to affect mortality, they increase hospitalization rates and risks of limb amputation [29]. Low levels of ATP and insufficient blood supply to peripheral nerves make these vulnerable to ischemia. Damaged nerve endings lead to pain perception because of disrupted action potentials, increased nodal Na+ currents, and altered K+ voltage-dependent channels that trigger neuronal excitability [30]. Hyperglycemia-induced metabolic abnormalities cause sensory, motor, and autonomic nervous system alterations. The features of sensory neuropathy include hyperalgesia, paresthesia, allodynia, and loss of protective sensations, with an elevated risk of reiterated trauma and unnoticed injuries [31]. Repeated minor trauma determines disintegration of the joints and overproduction of proinflammatory cytokines, such as tumor necrosis factor-α and interleukin (IL)-1β, promotes the expression of receptor of nuclear factor (NF)-ĸB ligand (RANKL), leading to osteoclast maturation. The main consequence is Charcot neuroarthropathy, commonly referred to as Charcot foot, a chronic and progressive degenerative arthropathy that has as hallmark deformities a “midfoot collapse”, hallux valgus, and loose bodies in the joint cavity. The main signs of motor neuropathy are atrophy of the small foot muscles, malposition of the toes, and foot deformities, such as claw toes and hammer toes, due to glycosylation of tendons that induces stiffness and shortening. The alteration in autonomic neuropathy reduces the function of sweat and sebaceous glands of the feet, resulting in dry skin and fissures. In this way, the skin becomes more susceptible to injury and infection because of the foot’s ability to moisturize. The intricate relationship among all these factors promotes the development and progression of ischemic ulcers, infectious diseases, and gangrene that characterize DF. Finally, some authors have also highlighted a relationship between DF and the gut microbiota. Three potential connections may exist between DF and the gut microbiota: firstly, the link between diabetic neuropathy and gut microbiome metabolites; secondly, the gut–skin axis; and thirdly, the use of antibiotic therapy in patients with infectious DF complications [32].

## 4. Mechanism of Action of SGLT2 Inhibitors

SGLT2-i are a class of medications crucial to the modern management of type 2 diabetes mellitus and heart failure. These drugs have been shown to reduce major cardiovascular events and hospitalizations in both diabetic and non-diabetic patients. They provide cardiovascular, nephroprotective, and neuroprotective benefits through their positive effects that engage multiple pathways currently under investigation. Understanding the detailed mechanism of action of these inhibitors will elucidate their effectiveness and highlight their multifaceted benefits beyond glycemic control [33].

In the kidneys, glucose undergoes filtration at the glomerulus and subsequent reabsorption in the proximal convoluted tubule (PCT). In humans, the transport maximum renal glucose reabsorption (TmG) capacity is 375 mg/min. For individuals with normal glucose tolerance, the filtration rate of glucose is approximately 180 g/day or 125 mg/min, assuming a normal glomerular filtration rate (eGFR) of 180 L/day and an average plasma glucose level of 100 mg/dL. This filtration rate is below the TmG capacity, allowing for complete reabsorption of glucose and preventing glycosuria. In contrast, individuals with poorly controlled T2DM often experience a filtered glucose load that surpasses the TmG capacity, leading to glycosuria. Typically, in healthy individuals, glycosuria does not occur until blood glucose levels exceed approximately 180 mg/dL.

Glucose reabsorption in the PCT is facilitated by SGLTs. SGLT2, located in the S1 segment of the PCT, is a high-capacity, low-affinity transporter responsible for reabsorbing about 90% of the filtered glucose. SGLT1, situated in the S2 and S3 segments, handles the remaining glucose with high affinity but lower capacity. The Na/K ATPase pump on the basolateral membrane maintains a sodium gradient by extruding three sodium ions from the cell in exchange for two potassium ions, driving secondary active transport of glucose into the PCT cells. Glucose then exits the PCT cells into the bloodstream through GLUT1 and GLUT2 transporters on the basolateral membrane [34,35]. In patients with uncontrolled diabetes, the renal TmG capacity can increase by approximately 20%, potentially due to upregulated SGLT2 expression in the PCT. However, not all studies consistently prove this overexpression [36]. SGLT2-i work by lowering both the TmG and glucose reabsorption threshold, thereby inducing glycosuria. These inhibitors can also cause glycosuria in non-diabetic individuals by significantly lowering the glucose reabsorption threshold from 180 mg/dL to 40–80 mg/dL [37]. Despite SGLT2 reabsorbing the majority of filtered glucose, SGLT2-i typically increase urinary glucose excretion by around 80 g/day. Inhibition of SGLT2 leads to an increased glucose load reaching the SGLT1 transporter in the proximal tubule. Consequently, SGLT1 operates at its maximum reabsorption capacity (40%), which accounts for why less than 50% of the filtered glucose is excreted in the urine [38].

Patients treated with SGLT2-i show a modest but consistent reduction in blood pressure (BP), with systolic BP decreasing by 3–6 mmHg. This antihypertensive effect is due to multiple mechanisms. Firstly, SGLT2-i promote natriuresis by blocking sodium–glucose cotransport in the proximal tubule, addressing the reduced natriuresis and increased total body sodium seen in diabetic patients, which can lead to hypertension. Secondly, the osmotic diuresis caused by elevated glucose concentrations in the urine reduces plasma volume, contributing to lower BP. Thirdly, SGLT2-i inhibit the sodium–hydrogen exchanger 3 (NHE3) on the apical surface of renal epithelial cells, further reducing sodium reabsorption and enhancing natriuresis [39]. Additionally, SGLT2-i improve β-cell function, enhancing β-cell glucose sensitivity and function, as demonstrated with treatments like dapagliflozin and empagliflozin. This improvement, although not a direct effect on β-cells, likely results from the reduction in plasma glucose concentration and the amelioration of glucotoxicity [40,41].

## 5. The Effect of the SGLT2-i on Atherosclerosis

ATS is one of the main risk factors for cardiovascular diseases, which still today represent the leading cause of death worldwide [42,43]. For this reason, recognizing, preventing, and treating the conditions that increase vascular damage risk is fundamental to turning the course of atherosclerotic disease and the incidence of overall mortality and disability. Hence, introducing new drugs into clinical practice to treat atherosclerosis-related diseases may break new ground for reducing the impact of ATS in healthcare. T2DM is one of the main risk factors for ATS, as demonstrated by a higher incidence of the disease in diabetic patients compared with non-diabetic [44]. SGLT2-i were demonstrated to improve the inflammatory and oxidative state in diabetic patients, independently from the glycemic balance [45]. This result may be explained by the potential effect of these drugs on the immune and inflammatory pathways, resulting in a slowdown in atherosclerotic damage.

So, SGLT2-i reduce the progression of ATS in diabetic patients by reducing the plasma glucose levels and acting pleiotropically on the inflammatory processes.

The data supporting this hypothesis derive from recent studies evaluating the differences in the circulating levels of inflammatory proteins in SGLT2-i-treated and non-treated patients, demonstrating that SGLT2-i reduce the serum concentration of cytokines such as TNF-α, monocyte chemoattractant protein 1 (MCP-1), platelet endothelial cell adhesion molecule-1 (PECAM-1), vascular cell adhesion molecule (VCAM)-1, intercellular adhesion molecule 1 (ICAM-1), IL-1β, and IL-6 [43,44,45,46,47,48,49]. The basis for the reduction in proinflammatory cytokines was sought in experimental studies whose aim was to identify the molecular mechanisms of this effect. Several hypotheses have been proposed, such as the improvement in endothelial and vascular smooth cell dysfunction, reduction in body weight and arterial pressure, decrease in the recruitment of macrophages and foam cell formation, reduction in oxidative stress, and induction of autophagy [4,50,51].

### 5.1. Molecular Mechanisms of the Anti-Inflammatory Effect of SGLT2-i

#### 5.1.1. Improvement in Endothelial Function

Endogenous NO reduces vascular inflammation and ATS progression. SGLT2-i attend to preventing endothelial dysfunction, increasing the vasodilation induced by the higher bio-disponibility of NO through the activation of NO synthase phosphorylation [52,53] and the suppression of ROS in TNFα-induced endothelial cells (ECs) [54]. ECs express on their surface SGLT2 stimulated by angiotensin II, which provokes the intracellular activation of the nicotinamide adenine dinucleotide phosphate (NADPH) oxidases pathway, leading to ROS production [55,56]. Several studies have demonstrated that SGLT2-i reduce oxidative stress and ROS production by inhibiting the SGLT2/NAPDH oxidases pathway [57,58,59], contrasting the progression of atherosclerotic plaques. Moreover, SGLT2-i reduce the expression of adhesion molecules, such as VCAM-1 and intracellular adhesion molecule (ICAM)-1, which promote the recruitment of circulating monocytes into the vascular layer. Canagliflozin [60] and empagliflozin [45] have been shown to reduce VCAM-1 expression, while ICAM-1 is downregulated by luseogliflozin [61].

#### 5.1.2. Inhibition of NLRP3 Inflammasome

The inflammasome plays a crucial role in the atherosclerotic process by producing proinflammatory proteins such as IL-1 and IL-18 [62,63]. The role of the inflammasome was confirmed by the demonstration that its inhibition leads to the stabilization of atherosclerotic plaques [64] and, on the other hand, by the evidence that increased levels of NLPR3-mRNA have been found in the serum of patients affected with atherosclerosis compared with healthy controls [65,66]. SGLT2-i have been associated with suppressing the NLPR3 inflammasome with a consequent reduction in IL-1beta levels [67], as also demonstrated by the slowing of the atherosclerotic process in diabetic ApoE^−/−^ mice treated with these drugs [68]. On the one hand, the suppression of the inflammasome derives from lowering serum glycemia but also from direct and indirect mechanisms, independently from the hypoglycemic effect. Direct mechanisms comprise the reduction in oxidative stress, the activation of cellular autophagy, and the inhibition of the NF-kB signaling pathway.

ATS in diabetes is related to higher levels of ROS, which lead to migration and proliferation of the smooth muscle cells. Sukhanov et al. demonstrated that the use of SGLT2-Is reduced the expression of NLRP3 in smooth muscle cells expressing SGLT2 proteins and mRNA, reducing oxidative stress and the progression of aortic atherosclerosis under normal glycemic conditions [69]. Moreover, SGLT2-i can decrease the ROS–NLRP3–caspaspase-1 pathway, reducing the production of IL-1beta and IL-18 in macrophages, and countering the inflammation in the endothelium [68] and hepatocytic cells [70]. Dapagliflozin has been proved to be able to block NLRP3–caspase 1 signaling, reducing the production of IL-1beta in diabetic ApoE^−/−^ mice and T2DM rodent models [67,71]; empagliflozin blocks the proliferation of the smooth muscle cells inhibiting the production of IL-1beta and IL-18 acting in NLRP3–caspase 1 signaling [69], reducing the production of ROS responsible for the progression of vascular damage.

##### Upregulation of Autophagy

Autophagy is the terminal stage of cellular degradation in the NLPR3 inflammasome pathway. The failure in autophagy leads to the accumulation of damaged cellular residual and ROS, determining the further activation of NLPR3, and perpetuating the inflammation [72]. Starving-mimicking conditions, such as reducing glucose levels as a consequence of the glycosuric effect of SGLT2-i, can induce the activation of autophagy. SLGT2-i upregulates autophagy, restoring the phosphorylated AMPK activity and so reducing the NLPR3 inflammasome expression [73,74]. Empagliflozin [75] and dapagliflozin [73] showed the ability to induce autophagy restoration, suppressing AMPK/mTOR signaling in rat cardiac myoblasts and renal tubular cells, respectively. This effect was also confirmed in recent studies on animal models [75,76,77].

##### Inhibition of NFkB Signaling

**Figure 1:**
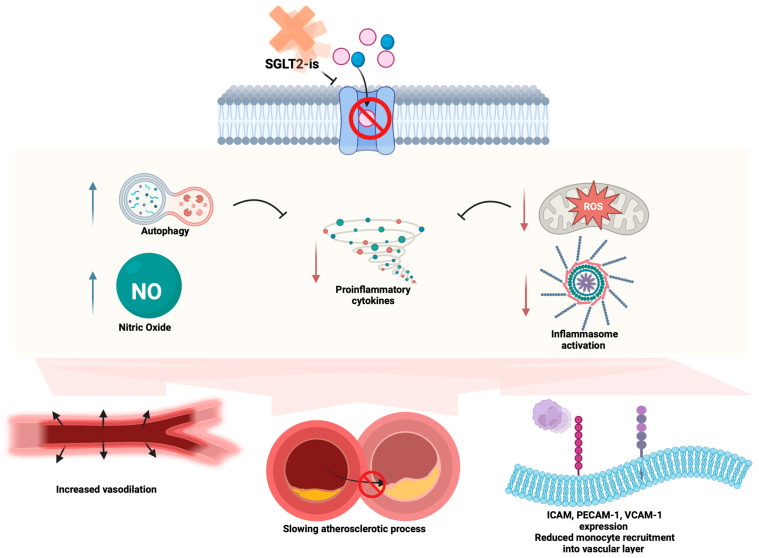
Molecular mechanisms of the anti-atherosclerotic effect of SGLT2-i. SGLT2-i reduce the expression of adhesion molecules, such as vascular cell adhesion molecule (VCAM)-1 and intracellular adhesion molecule (ICAM)-1, which promote the recruitment of circulating monocytes into the vascular layer, suppress NLPR3 inflammasome with consequent reduction in IL-1beta levels, and upregulate autophagy, restoring the phosphorylated AMP-activated protein kinase (AMPK) activity. (Created with BioRender.com).

The increase in intracellular glucose induces the expression of the proinflammatory molecule HMGB1 responsible for the activation of the NFkB pathway [78], leading to the production of proinflammatory cytokines (TNF-a, IL-1, and IL-6), monocyte chemotactic proteins, and adhesion protein molecule expression responsible for NLRP3 activation [79]. In this concern, SGLT2-i, by lowering the intracellular glucose levels, may be useful in reducing inflammation. Moreover, Abdollahi et al. demonstrated that the use of SGLT2-i reduced the expression of TLR4 on the cellular surface, reducing the activation of NLRP3 and the production of proinflammatory cytokines [80]. The anti-NFkB effect of SGLT2-i has been confirmed by the evidence of the decreased concentrations of IL-1b, IL-6, and TNF-a expression in the proximal tubular renal cells in diabetic models [73]. Finally, by reducing the glucose levels, SGLT2-i stimulate lipolysis, inducing b-hydroxybutyric acid (b-OHB) production, which acts by reducing the NLPR3 inflammasome, taking part in the anti-inflammatory role of SGLT2 indirectly [81,82]. In a recent small trial, Kim et al. demonstrated NLRP3 suppression and lower IL-1b and TNF-a levels in diabetic patients treated with SGLT2-i compared with sulfonylurea treatment, irrespective of the glycemia value [67]. The abovementioned mechanisms are summarized in Figure 1.

#### 5.1.3. Inflammaging and the Cellular Senescence

The term inflammaging has recently been introduced to describe a low-grade proinflammatory state occurring during aging, which concurs with vascular senescence [83,84]. The leading determinant in inflammaging is IL6, and recent studies have shown that SGLT2-i positively affect the aging process [85], reducing the circulating IL-6 levels [86]. Adenosine 50-monophosphate-activated protein kinase (AMPK) and sirtuin-1 are the two mediators involved in aging and the cellular metabolic process [87]. AMPK is a metabolic regulator and works as an energy sensor in inflamed macrophages, reducing anabolism and promoting catabolism and ATP production [88]. It is activated by an increased adenosine monophosphate (AMP)/adenosine triphosphate (ATP) ratio; moreover, AMPK upregulates sirtuin-1 while having an inhibitory role in the mTOR pathway [89]. The activation of AMPK has a protective role from oxidative stress, apoptosis, mitochondrial dysfunction, and proinflammatory state [90]. SGLT2-i increase AMP levels, restoring the AMP/ATP balance through AMPK activation [91]. This effect was demonstrated in clinical studies showing that AMPK phosphorylation induced by the administration of SGLT2-i was linked to the amelioration of cardiovascular function in patients who underwent cytostatic treatment [75,92]. Moreover, AMPK increases the NAD+ levels, which is a potent activator of sirtuin-1 [90]. Sirtuin-1 is involved in lipolysis, augments insulin sensitivity, and limits proinflammatory macrophage activity, detecting the body’s bioenergy demands. Chronic inflammation is related to sirtuin-1 suppression, inducing the progression of vascular damage. Thanks to the glycosuric effect of SGLT2-i and the induction of nutrient deprivation, the use of this medication can activate sirtuin-1/PGC-1a/FGF21 signaling, contributing to the cardiovascular protective action [93,94]. Several studies have supported the role of SGLT2-i on these molecular patterns [93,95]. Empagliflozin has been shown to reduce the gene expression of IL-6, IL-1β, TNF-α, and monocyte chemoattractant protein-1 (MCP-1) mRNA, which induces the upregulation of AMPK in macrophages and reduces the expression of NF-κB. Acting on these pathways, empagliflozin has demonstrated a direct anti-inflammatory role in atherosclerotic plaque progression [95]. Furthermore, the AMPK/SIRT1 pathway appears to be suppressed by adiposity and high glucose levels, which results in the inactivation of this intracellular pathway and an increase in the inflammatory response. Dapagliflozin can restore the intracellular SIRT1, PGC-1α, and p-AMPK in the human umbilical vein endothelial cells exposed to high glucose concentrations, demonstrating its role in preventing vascular damage, reducing oxidative stress, inflammation, and cellular death [93]. The accumulation of senescent cells participates in the aging and development of age-related diseases, and ATS appears to be influenced by the accumulation of these cells into vascular plaques. The elimination of the senescent cells, known as senolysis, has been related to improving cardiovascular disease, metabolic dysfunction, and the incidence of neoplastic disease [96,97]. Due to this effect, senolysis may be the target for new therapy aiming to change the course of atherosclerotic disease. Some recent studies have proven the role of SGLT2-i in eliminating senescent cells [98]. A recent study carried out by Katsuumi et al. tried to explain the mechanisms through which SGLT2-i induce senolysis [99]. In a high-fat diet mice population, the administration of canagliflozin resulted in the reduction in senescence-associated β-galactosidase activity and decreased expression of negative cell cycle regulators compared with the control group after one week of treatment, while after four weeks of treatment, a reduction in the expression of proinflammatory senescence-associated secretory phenotype factors was taken over. These results support the hypothesis of a potential role of SGLT2-i in senescence. Senescent cells activate the immune system by producing senescence-associated secretory phenotype factors, which induce chronic inflammation [100]. In healthy people, it leads to the elimination of the damaged cells. However, this mechanism is impaired in senescent cells where the accumulation of SAPS factors leads to the expression of PD-L1, contrasting the removal systems [101]. AMPK suppresses PD-L1 signaling; hence, SGLT2-i have been proposed as a potential inducer of senolysis by suppressing PD-L1 indirectly. In their study, Katsuumi et al. [99] have found an increase in PD-L1 expression in the gonadal white adipose tissue of high-fat diet-fed mice and, interestingly, this expression was reduced after a short-term canagliflozin treatment, and the use of SLGT2i increased the recruitment of NK cells and CD8+ T cells. These effects were independent of the impact on the glycemic status. There was no difference in the body weight, glucose levels, or lipid profile between the canagliflozin-treated and the control groups.

#### 5.1.4. Effect on Mitochondrial Function

Dysfunction of mitochondria is associated with the accumulation of ROS and is related to cellular senescence [102]. Cells have developed antioxidant mechanisms concerning the control of the activity of enzymatic systems (NAD, NADH oxidase, xanthine oxidase, cyclooxygenases, and monooxygenases) involved in ROS production [103]. SGLT2-ii were shown to affect mitochondrial function, preventing cellular dysfunction and ROS-derived damage. This role is explicated through several mechanisms identified in cellular studies. On the one hand, SGLT2-i reduce the mitochondrial concentrations of calcium and the availability of electron donors (such as NADH), decreasing ROS production and oxidative stress in endothelial cells, and preventing microvascular damage in diabetes mellitus [104]. Moreover, SGLT2-i reduce NADPH oxidase (NOX)4 protein expression, whose activity is related to the increase in ROS production. Both canagliflozin [105] and empagliflozin [106] showed this effect, ameliorating the myocardial function in mice models.

In a recent study, Pignatelli et al. [107] have shown a reduction in NOX2 activity and H_2_O_2_ levels in diabetic patients after 15 days of treatment with SGLT2-i; furthermore, the use of SGLT2-i has demonstrated the improvement in platelet function through the reduction in thromboxane, soluble P-selectin, and soluble CD40 ligand levels, suggesting a further mechanism that takes action in preventing the progression of ATS.

Finally, SGLT2-i normalize the mitophagy process via the transcription factors PGC-1α and mitochondrial transcription factor A and by regulating mitochondrial fission via dynamin-related protein 1, mitofusin 1, and mitofusin 2 and increasing ATP production, preserving the mitochondria function and reducing the risk of cardiovascular complications in diabetes mellitus [104,108].

### 5.2. The Impact of SGLT2-i in Subclinical and Clinical Atherosclerosis

From these previous data, clinical evidence has been searched to analyze the impact of SGLT2-i on vascular diseases in vivo. The initial signs of endothelial dysfunction are the carotid intima-media thickness and pulse wave velocity (PWV), which are markers of subclinical atherosclerosis. In 2022, Hodrea et al. [109] carried out a study on the metabolic effect of dapagliflozin in a population of the murine model in which type 1 diabetes mellitus was inducted. The study showed that the use of dapagliflozin prevented aortic IMT, as demonstrated by the histological finding of the prominence of wavy internal elastic lamina in the control rats compared with the treated models.

A randomized parallel trial (the UTOPIA study) [110] conducted on a population of 340 diabetic patients with no history of CVD showed that the administration of tofogliflozin had a beneficial effect on subclinical atherosclerosis. After 104 weeks of treatment, there was a significant reduction in the carotid intima-media thickness, even if no differences were noted compared with the conventional treatment, while there was a significant reduction in PWV in tofogliflozin-treated patients compared with the conventional therapy. A prospective observational extension of the study [111] for a further 104 weeks was conducted to evaluate the change in IMT-CCA related to long-term treatment as the primary endpoint. Moreover, other secondary endpoints were set as indicators of vascular risk factors, such as changes in HbA1c, lipids, serum creatinine, and urinary albumin excretion, and biomarkers of arteriosclerosis such as the ankle–brachial index (ABI) and brachial–ankle pulse wave velocity (baPWV). The long-term treatment with tofogliflozin confirmed the previous results regarding the change in IMT-CCA, with a significant reduction in the intima-media thickening but without significant differences with the control group. On the other hand, the difference in the mean baPWV values was maintained during the follow-up period between the two groups, showing that tofogliflozin induced long-term attenuation of baPWV progression and positively affected arterial stiffness over almost four months. Given that IMT and baPWV are both indicators of subclinical atherosclerosis, the difference in these studies’ results may be explained by the different stages of arterial damage. IMT reflects a structural change in the vascular layer, suggesting an advanced stage of the atherosclerotic process, while PWV is a marker of impaired elasticity due to functional changes. Moreover, the study showed a significant improvement in the HbA1c and HDL-C levels, BMI, waist circumference, and systolic blood pressure in the tofogliflozin-treated group compared with the conventionally treated group, while no differences were found regarding the change in renal function between the groups. The above results were also found in a previous study, which demonstrated a reduction in the PWV in 160 diabetic patients who underwent SLGT2-i therapy for 12 months compared with insulin treatment [112]. Kourtidou et al. used in their study the “pulse wave velocity”, “augmentation index”, and the “augmentation index adjusted to a heart rate of 75 beats/min” as markers of arterial stiffness, which were significantly reduced in the group of diabetic patients treated with SGLT2-i compared with the control group [113]. Moreover, a recent study demonstrated a pivotal role of SGLT2-i in plaque stabilization. Dapagliflozin has shown the capability to reduce lipid accumulation on atherosclerotic plaques and increase the cap-to-lesion height ratio and the collagen content in a population of diabetic murine models, while no effect was demonstrated regarding the change in the plaque size [114]. These data suggest the possible role of dapagliflozin on the fibrosis and remodeling of plaques rather than on inflammation. The effect of SGLT2-i on plaque composition has also been investigated in humans. Sardu et al. demonstrated that the use of SGLT2-i increased the fibrous cap thickness in a cohort of diabetic patients with coronaropathy, reducing the risk of major adverse cardiovascular events (MACEs) as a result of less vulnerability of the coronary lesions [115]. Finally, given the role of the assessment of the coronary artery calcification score in determining cardiovascular risk, more studies may be provided to evaluate the possible role of SGLT2-i in determining calcium score changes, but studies in this field are not currently available.

## 6. Molecular Effect of SGLT2-i on Neuropathy

Diabetic peripheral neuropathy (DPN) represents a severe microvascular condition that affects about half of the patients with T2DM, targeting the peripheral nervous system’s sensory, motor, and autonomic neurons. DPN also affects diabetic patients despite adequate glycemic control, resulting in high morbidity. Nevertheless, there are just a few studies in the literature on the molecular effects of these drugs in diabetic neuropathy.

**Figure 2:**
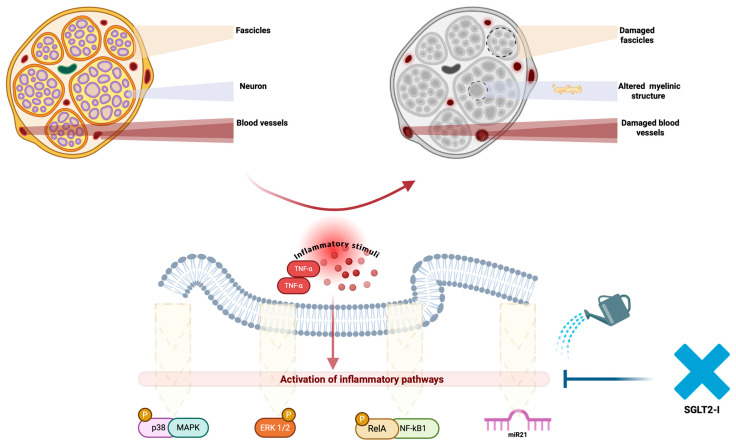
Molecular effect of SGLT2-i on neuropathy. SGLT2-i inhibit the signaling pathways involving p-p38 MAPK, p-ERK1/2, p-NF-κB p65, IL-1β, and TNF-α, reduce miR-21, and increase RECK expression, alleviating neuropathic damage. (Created with BioRender.com).

A recent study by Abdelkader et al. [116] explored the potential of empagliflozin, to enhance streptozotocin-induced DPN in rats with information on its precise signaling mechanism. In their study, it was observed that while EMPA did not regulate blood glucose levels or body weight, it effectively halted oxidative stress by enhancing SOD activity and decreasing lipid peroxidation. EMPA also elevated p-AMPK levels, improved the ATP/AMP ratio, and inhibited the signaling pathways involving p-p38 MAPK, p-ERK1/2, p-NF-κB p65, IL-1β, and TNF-α. Importantly, EMPA reduced miR-21 and increased RECK expression, leading to decreased matrix metalloproteinase activity. Consequently, the beneficial effects of EMPA on DPN are likely due to mechanisms independent of glucose regulation (Figure 2). Specifically, EMPA mitigated energy deprivation and enhanced AMPK phosphorylation, which plays a role in improving DPN by targeting various signaling molecules. EMPA-induced AMPK activation in the sciatic nerve suppressed mitogen-activated protein kinase (MAPK) signaling pathways crucial for neuropathic pain through the sensitization of peripheral nociceptors and diminished plasticity of related proteins [117]. AMPK activation negatively regulates p38 MAPK and extracellular signal-regulated kinases (ERK) 1/2 by phosphorylating adaptor proteins involved in receptor tyrosine kinase regulation and inhibiting upstream activators like small GTPases [118]. Inhibition of p38 MAPK or ERK has been shown to alleviate neuropathic damage. Additionally, after nerve injury, p38 MAPK/ERK1/2 upregulated the transcription factor NF-κB p65 and increased downstream mediators such as IL-1β and TNF-α, which contribute to pain hypersensitivity [119]. EMPA mitigated the negative effects of these inflammatory mediators by suppressing the expression of p-p38 MAPK, p-ERK1/2, and p-NF-κB p65. mTOR has been implicated in the development of DPN in STZ-treated animals due to reduced AMPK activation or increased TNF-α levels. This could be explained by reduced levels of APPL1, a protein critical for synaptic plasticity, or by mTOR activation. Mechanical and thermal hyperalgesia may be driven by synapsin II-mediated neurite growth [120]. Conversely, EMPA improved hyperalgesia through its effects on AMPK and anti-inflammatory properties. Furthermore, mTOR inhibition by EMPA may enhance DPN management through autophagy induction, which helps remove damaged cellular components and allows cells to meet their metabolic needs, thus increasing myelin sheath thickness and myelinated axons [121]. Inhibition of mTOR also promotes ULK1 phosphorylation and beclin-1 activation, which alleviates pain by enhancing autophagy [122]. Elevated levels of MMP-2 and MMP-9 were found in the sciatic nerves of diabetic rats, in an interesting study by Moustafa et al. [123], who reported that controlling extracellular matrix remodeling can alleviate DPN. This increase is linked to heightened oxidative stress and p38 MAPK/NF-κB p65 pathways affecting MMP regulation. EMPA’s effect on reducing MMP expression may be due to increased RECK levels, an endogenous MMP inhibitor, through its inhibition of oxidative stress, p-NF-κB p65, and p-p38 MAPK/miR-21 pathways. Additionally, EMPA treatment reversed the effects of miR-21 stimulation on pain thresholds, as documented in nerve injury models [124]. Overall, the beneficial effects of EMPA were nearly abolished by dorsomorphin, an AMPK antagonist, highlighting the critical role of AMPK in managing DPN. Wang et al. also demonstrated that combining dapagliflozin with mecobalamin significantly improves clinical symptoms in patients with type 2 diabetes peripheral neuropathy. This combination therapy not only reduces blood glucose levels but also effectively regulates changes in MDA, SOD, and COX-2 levels, leading to reduced nerve cell damage. Additionally, it enhances the conduction velocities of both sensory and motor nerves [125]. Additionally, available data suggest that gliflozins could offer benefits in T2DM, ATS, and cognitive decline through various mechanisms, such as anti-inflammatory and anti-atherosclerotic effects, SGLT1 inhibition, AChE inhibition, reduction in oxidative stress, cerebrovascular remodeling, and restoring metabolic balance. However, long-term clinical studies are necessary to determine the clinical relevance of these mechanisms, as the athero-protective and neuroprotective effects of SGLT2-i will require extended use to become evident [126].

## 7. Immunomodulation and SGLT2-i

Diabetes is a metabolic condition that is caused by inflammation as a result of the complex immunological process. Insulin resistance leads to hyperglycemia due to a series of immune responses that amplify the inflammatory state. The immune system in diabetic individuals is weakened by defects in the innate immune response and dysfunction in the adaptive immune response, leading to an increased vulnerability to invading pathogens. In addition to cellular immunity weakness, neuropathy can result in natural, barrier damage. Numerous studies have been conducted to determine the diabetes-related mechanisms that impair the host’s defense against pathogens. These mechanisms include suppression of cytokine production, defects in phagocytosis, dysfunction of immune cells, and failure to kill microbials. Chronic hyperglycemia has been found to cause impairment of phagocytosis through defects in complement receptors and Fcγ receptors on isolated monocytes, as demonstrated by Restrepo et al. [127]. Berrou et al. showed that NK cells, which have the importance of controlling invading pathogens, were ineffective [128]. Joshi et al. reported that neutrophil action to produce neutrophil extracellular traps was suppressed during hyperglycemia, leading to susceptibility to infections [129]. As a result, individuals with diabetes are commonly more susceptible to infections. The prevalence of T2DM will lead to a rise in the prevalence of infectious diseases and related comorbidities. The risk of death from infection for T2DM individuals was significantly higher than for healthy individuals (pneumonia 70%, sepsis 7%, lower respiratory tract infections 5%) [130]. The burden of diabetes and its complications is still present globally, as evidenced by the significant increase in mortality and morbidity each year, as reported by the World Health Organization’s health statistics. Lower respiratory tract infections, such as pulmonary tuberculosis and pneumonia, urinary tract infections, and skin and soft tissue infections, are more likely to occur in individuals with diabetes, according to several studies. Interestingly, treatment with empagliflozin is demonstrated to elevate neutrophil counts and enhance their functionality by inhibiting apoptosis, restoring phagocytic activity and chemotactic response, normalizing the oxidative burst, and stabilizing both cellular and plasma levels of defensins and lactotransferrin [131]. For this reason, SGLT2-i are considered an excellent alternative to treat the neutropenia present in G6PC3-deficiency [132].

Foot ulcers, which are often infected, are a common occurrence in diabetic patients. Fournier’s gangrene is a condition that occurs rarely [133]. Diabetes is often accompanied by impaired wound healing, which is the most common and significant complication. According to estimates, 25% of people with diabetes will experience foot ulcers throughout their lifetime. In the first stages of wound healing, inflammatory cells, such as cytokines, neutrophils, and chemokines, are recruited. However, this process of recruitment is delayed. Initiating wound healing is crucially aided by proinflammatory cytokines, which increase IL-1ß, TNF-a, IL-6, TGF-ß, and CRP, and decrease IL-10. As a result, the wound environment is unfavorable for the healing process. Lower concentrations of TNF-a, a proinflammatory cytokine, can initiate inflammation during the inflammatory phase of wound healing. Increasing the concentration can inhibit the process of wound healing by degrading the extracellular matrix formation during the remodeling phase of wound healing [134]. The risk of developing foot ulcers is high among individuals with diabetes. Infectious wounds are common, and diabetic foot ulcers that do not heal can lead to the amputation of part or all of the foot or even the lower leg. According to a meta-analysis, 20% to 58% of patients develop another ulcer within one year of wound healing. The rate of amputation was 2.23 times higher for patients with recurrent foot ulcers than those with first-time foot ulcers [135]. Very few studies have investigated the modulating effect of SGLT2 on the immune system. Substantially, the immunomodulatory action of SGLT2-i can be simplified based on the effects on two types of cells: macrophages and T lymphocytes. Some studies have analyzed the effect of SGLT2-I in wound healing [136]. Particularly, dapagliflozin may promote neovascularization in diabetic mice via HIF-1α-mediated enhancement of angiogenesis inducing pluripotent stem cells (iPSC)-EC NVs and facilitating diabetic wound healing by the HIF-1α/VEGFA pathway.

It is interesting to notice that the pleiotropic and immunomodulatory effects of SGLT2-i are also evident in the known cardioprotective effects and reduction in atrial fibrillation onset in diabetic patients [137]. Multiple markers of inflammation are elevated in AF and to predict responses to treatments of AF including anti-arrhythmics and cardioversion. It is possible that the immunomodulatory effect of SGLT2-i also occurs at the cardiac level, thus reducing the onset of atrial fibrillation. Finally, some studies have demonstrated significant reductions in body mass index, weight, waist and hip circumference, and body fat percentage in both groups. In contrast, the authors showed a significant increase in muscle mass percentage [138].

### 7.1. Macrophage Polarization

Macrophages have two phenotypes, and their polarization controls the inflammatory state. M1-macrophages have a proinflammatory role, while M2-macrophages have an anti-inflammatory function, producing cytokines such as IL-10 and IL-1 receptor antagonist. M1/M2 polarization is a reversible process depending on the lipid and glucose concentrations: in obese patients, the M2-produced cytokines are decreased, and the proinflammatory state prevails. Empagliflozin reduces M1-macrophage expression and induces M2-polarization, achieving a protective role in the inflammation in the white adipose tissue and liver macrophages, as demonstrated in one study carried out on high-fat obese mice [139].

### 7.2. T Cell Modulation

Moreover, since T cells minimally express SGLT2 on their surface, SGLT2-i have an effect on reprogramming the T cell response. Jenkins et al. [140] demonstrated that exposure to canagliflozin impairs ERK and mTORC1 activity and inhibits the mitochondrial glutamate dehydrogenase, altering the T cell function. Furthermore, this study showed that canagliflozin reduced the production of inflammatory proteins by the CD4 cells from patients affected with autoimmune diseases, suggesting the possible role of these medications in the modulation of immune and autoimmune responses. Empagliflozin was shown to increase the regulatory T cell subsets, reducing the proinflammatory Th1 and Th17 subsets in immune thrombocytopenia patients, and modulating the CD4 response through the mTOR signaling pathway [141].

## 8. SGLT2-i and Peripheral Artery Disease

The medical management of PAD in patients with diabetes does not differ from that recommended for patients with atherosclerotic cardiovascular diseases in general, including SGLT2-i. The potential of SGLT2-i to improve peripheral artery disease (PAD) outcomes lies in their ability to address multiple cardiovascular risk factors simultaneously. By reducing blood pressure, improving endothelial function, and exerting anti-inflammatory effects, SGLT2-i could slow the progression of ATS in PAD, reducing the incidence of stenosis, critical limb ischemia, and the need for revascularization procedures. Although direct evidence specifically targeting PAD is less abundant compared to coronary artery disease, emerging studies indicate potential positive outcomes. Large-scale cardiovascular outcome trials have established the efficacy of SGLT2-i in reducing MACEs in patients with T2DM. The EMPA-REG OUTCOME trial evaluated the effects of empagliflozin on cardiovascular outcomes in patients with T2DM and documented cardiovascular disease. The study demonstrated a significant reduction in MACEs, cardiovascular death, and hospitalization for heart failure. Although PAD-specific outcomes were not the primary focus, the overall cardiovascular benefits suggest potential positive effects on PAD patients [142]. The DECLARE-TIMI 58 trial assessed dapagliflozin in patients with T2DM, including those with multiple cardiovascular risk factors and established cardiovascular disease. The results showed a significant reduction in heart failure hospitalization and a favorable trend in reducing cardiovascular death. Although PAD-specific data were limited, the trial’s findings support the overall cardiovascular protective effects of SGLT2-i [143]. The CANVAS program, which investigated canagliflozin, also reported a reduction in MACEs, even if there was an initial observation of an increased risk of lower-limb amputations [144]. This finding has not been confirmed in the CREDENCE trial comparing canagliflozin with placebo in patients with T2DM and chronic kidney disease, nor cardiovascular outcomes with other SGLT2-i. Furthermore, subsequent analyses indicated that the cardiovascular benefits, including reduced hospitalization for heart failure and improved renal outcomes, may outweigh the risks, especially with careful patient selection and monitoring. Other studies assessed the prevalence of SGLT2-i treatment and its association with restenosis risk in patients with diabetes mellitus undergoing endovascular therapy for symptomatic peripheral artery disease. SGLT2-i were rarely used in patients with T2DM who underwent femoropopliteal endovascular therapy using a drug-coated balloon for symptomatic peripheral artery disease in real-world settings. SGLT2-i treatment was not associated with an increased risk of restenosis [145]. Mavrakanas et al.’s meta-analysis [146] focused on cardiovascular and renal outcomes with SGLT2-i, finding consistent reductions in adverse cardiovascular events. The analysis suggested that the improved endothelial function and reduced arterial stiffness associated with SGLT2-i could potentially lower the incidence of restenosis and the need for subsequent revascularization procedures in PAD patients. Furthermore, in a longitudinal, national cohort of patients with T2DM, the use of SGLT2-i was compared with dipeptidyl peptidase-4 inhibitors (DPP4i). SGLT2 inhibitors were found to have similar risks of ischemic stroke and acute myocardial infarction but were associated with a lower risk of lower-limb ischemia requiring revascularization [147].

However, there is currently a substantial gap in evidence for this population, largely relying on post hoc analyses or real-world studies [148]. A meta-analysis by Chu Lin et al. (2019) reviewed data from randomized controlled trials involving SGLT2 inhibitors in patients with T2DM. The analysis examined the relationship between changes in hemodynamic status (such as diuresis and blood pressure reduction) and the risk of lower-limb complications. It found that greater reductions in body weight and blood pressure might be linked to an increased risk of lower-limb complications in SGLT2-i users. These complications included arterial thrombosis, arterial stenosis, ischemic limb pain, extremity necrosis, and intermittent claudication [149]. In a recent meta-analysis, comparisons were made between individuals with type 2 diabetes treated with SGLT2-i and those receiving control treatments. The primary outcomes assessed were acute coronary syndrome, PAD, and ischemic stroke, while secondary outcomes included cardiovascular mortality and all-cause mortality. Risk ratios and 95% confidence intervals (CIs) were calculated using a fixed-effects model. The validity of each study meeting the inclusion criteria was evaluated using Cochrane’s risk-of-bias (RoB2) tool. Subgroup analysis indicated that none of the individual SGLT2-i (canagliflozin, dapagliflozin, empagliflozin, and ertugliflozin) had a significant impact on outcomes when analyzed separately [150]. There are some discussions as to whether the use of GLP-1 RAs could be preferable in patients with lower-extremity atherosclerotic disease. Ongoing studies may help to clarify this question in the future. SGLT2-i have demonstrated significant cardiovascular benefits in patients with T2DM, including those with high cardiovascular risk profiles that overlap with PAD. While direct evidence specifically targeting PAD is limited, existing data from major cardiovascular outcomes and meta-analyses support the potential for SGLT2-i to reduce vascular events in this population. Nevertheless, SGLT2-i have shown beneficial effects on arterial stiffness [151] and micro- and macrovascular endothelial function [152], which may give hope for the potential slowing of PAD progression in diabetic subjects.

Further dedicated studies are needed to confirm these findings and optimize the use of SGLT2-i in PAD patients, ensuring careful monitoring to mitigate any potential risks.

## 9. SGLT2-i and Risk of Infection

The SGLT2 transporter is responsible for the reabsorption of more than 90% of renal glucose in the urine filtered by renal glomeruli. However, post-marketing adverse event reports have raised concerns about the safety of SGLT2-i, including adverse effects such as genital and urinary tract infections, amputation, and diabetic ketoacidosis. SGLT2-i reduce glucose levels by decreasing renal glucose reabsorption and increasing glucose excretion in urine, which seems to be a favorable setting for bacteria growing. Glucosuria was considered to be a more likely risk factor [153].

The primary factors that contribute to urinary tract infection (UTI) in diabetes are prolonged illness, suboptimal glycemic control, an inadequate immune system, diabetic microangiopathy, autonomic neuropathy, urinary tract abnormalities, neurogenic bladder, and recurrent vaginal infections [154]. Data from a meta-analysis related to all infections showed that SGLT2-i are associated with a significant threefold increased risk of genital tract infections compared to placebo and a fourfold increased risk compared to other antihyperglycemic agents. This meta-analysis did not find a significant association between SGLT2-i and UTI risk compared to placebo or other anti-diabetes agents, unlike previous studies. Individual drug analyses revealed a significant increase in the risk of UTI with dapagliflozin 10 mg daily, but not with canagliflozin, empagliflozin, or dapagliflozin 5 mg daily. The risks of urosepsis or pyelonephritis were not increased by SGLT2-I [154,155].

In December 2015, the Food and Drugs Administration issued a warning for SGLT2-i due to the possibility of severe UTIs, following reports of severe sepsis and pyelonephritis in patients using these drugs. However, the most recent data do not reveal an association between SGLT2-i and UTIs. A meta-analysis revealed that non-diabetic individuals taking SGLT2-i had, to a lesser degree, an increased risk of genital infections and urinary tract infections [156,157,158,159,160].

In January 2022, the American Diabetes Association found 491 instances of Fournier’s gangrene that were linked to SGLT2-i. Fournier’s gangrene is a rare progressive adverse reaction. Soft tissue structures can be penetrated by Fournier’s gangrene, and subcutaneous fat and muscle can be destroyed, resulting in necrosis of perineal, perianal, and genitourinary areas. The death rate in some instances can reach up to 50%. It is not yet clear whether SGLT2-i cause Fournier’s gangrene, but it could be linked to the stimulation of urinary glucose excretion. A high concentration of urinary glucose can create an ideal environment for bacterial growth, thereby increasing the risk of gangrene. In this study, cultures showed that patients with Fournier’s gangrene were monocultures or a combination of bacteria of aerobic, anaerobic, or facultative anaerobic bacteria [161]. Unfortunately, most of the data on the infectious risk in diabetic patients treated with SGLT2-i concern genitourinary tract infections. Little or nothing has been studied about the effects of SGLT2-i on soft tissue infections, especially of the lower limbs, in diabetic patients. The overall benefits and risks of SGLT2-i for managing T2DM need to be taken into an estimate when evaluating them. Because of their many advantages, SGLT2-i have become the primary treatment for T2DM, and their usage is expected to explode in the coming years. Controversy exists over the risk of infection while taking SGLT2-i. Different meta-analysis findings regarding the correlation between SGLT2-i and the risk of all infections did not indicate a significant association between SGLT2-i and UTI risk compared to placebo or other anti-diabetes agents. However, several studies showed a significant threefold increased risk of genital tract infections compared to placebo and a fourfold increased risk compared to other antihyperglycemic agents. In an interesting meta-analysis [155], the risk of UTIs was not increased with SGLT2 inhibitors compared to placebo. They were associated with a reduced risk of gastroenteritis but did not affect the risk of respiratory tract infections showing a safe profile.

Only selected vulnerable patients who are more susceptible to genital infections and UTIs (like those with suboptimal glycemic control, urinary tract abnormalities, neurogenic bladder, recurrent vaginal infections, perineal gangrene, recurrent bladder infections, and neurological bladder) may benefit from avoiding the use of SGLT2-i.

## 10. SGLT2-i and Neuropathy

The cardioprotective and nephroprotective effects of SGLT2-i are well documented and strongly supported by numerous studies. However, although the evidence is still limited and requires further investigation, particularly regarding the long-term use of these medications, their neuroprotective effects should also be emphasized. Wang et al. [125] investigated the effect of dapagliflozin combined with mecobalamin on blood glucose concentration and serum MDA, SOD, and COX-2 as well as the change in nerve conduction velocity [motor nerve conduction velocity of median and common peroneal nerve and sensory nerve conduction velocity before and after treatment. The results indicated that both the motor nerve conduction velocity and sensory nerve conduction velocity in the median and common peroneal nerves were significantly higher in the study group compared to the control group. This demonstrates that combining dapagliflozin with mecobalamin can notably enhance nerve conduction velocity and alleviate clinical symptoms in patients with DPN. In a three-year follow-up study, Ishibashi et al. [162] aimed to demonstrate whether SGLT2-i offered protection against diabetic neuropathy and nephropathy in patients with T2DM by reducing the variability in glycemia and extra glycemic factors or their average. In particular, regarding neuropathy, they demonstrated that the mean Z-scores of eight neurophysiological tests (median motor nerve conduction velocity and amplitude, sural sensory nerve conduction velocity and amplitude, vibration perception threshold, coefficient of variation of R-R interval, warm perception threshold, and cold perception threshold) at baseline and endpoint in two diabetic cohorts were significantly lower compared to the control subjects. SGLT2-i treatment significantly increased the mean Z-score, while it deteriorated in patients without SGLT2-i. Kandeel conducted a meta-analysis to assess the benefits of using SGLT2-i to manage DN [163]. It emerged that SGLT2-i could be neuroprotective in patients with DN by considerably increasing the sensory and motor nerve conduction velocity, improving clinical manifestations of DPN, and reducing sympathetic nervous system activity.

## 11. Risk of Amputation

T2DM is often accompanied by micro- and macrovascular complications. DFUs are the most common cause of hospitalization among diabetic patients. DFU is the cause of approximately 80% of nontraumatic lower-limb amputations worldwide. Estimates suggest that a lower-extremity amputation occurs every 20 s for a person with diabetes. According to previous studies, amputations resulting from diabetes have a high mortality rate, with a 5-year survival rate of 41% to 48%. To decrease the likelihood of lower-extremity amputation and other microvascular events, diabetes therapy focuses on maintaining optimal glucose control [135,164,165,166]. In 2013, the U.S. Food and Drug Administration approved SGLT2-i as antihyperglycemic agents for T2DM. SGLT2-i, including canagliflozin, dapagliflozin, and empagliflozin, work by inhibiting renal glucose reabsorption, which leads to an increase in glucose excretion and a decrease in plasma glucose. Clinical benefits include weight loss and reduced risk of major cardiovascular events, heart failure, and all-cause death. Although there are potential benefits, the Food and Drug Administration issued a Drug Safety Communication in 2017 stating that canagliflozin raises the risk of leg and foot amputation. Safety concerns were raised by the CANVAS program due to a significant increase in the risk of total amputation. However, the European Medicines Agency included warnings about the danger of lower-limb amputation in the product information of every drug in the class. On the other hand, in Japan, a warning is added to the label of canagliflozin. Recently, several observational studies have sought to corroborate this finding in broader populations, with mixed conclusions [167]. The relationship between SGLT2-i and amputation risk remains unclear, but there are possible pathways that involve volume depletion and reduced tissue perfusion [168]. SGLT2-i decreases plasma glucose by raising the renal threshold for glucose uptake, resulting in increased urinary glucose excretion and osmotic diuresis, which may be linked to a decrease in intravascular volume. A meta-analysis suggests that the decrease in body weight and blood pressure in SGLT2-i users could be indirectly attributed to the loss of body fluid and the change in hemodynamic status. The hypothesis that the relationship between SGLT2-i and amputation risk is based on hypovolemia is validated by the finding that diuretics increase the risk of lower-limb amputation in patients with T2DM. Previously, it was reported that patients with T2DM taking thiazide diuretics have an increased risk of lower-extremity amputation. Volume depletion caused by diuretics may cause circulatory failure in the distal peripheral arterial beds and increase insufficient perfusion in the extremities. In addition, another meta-analysis indicated that SGLT2-i users had a significantly higher risk of amputation, PAD, and DF when their systolic and diastolic blood pressures were reduced. It was found that patients using SGLT2-i had a slight increase in the risks of amputation and PAD, primarily due to the use of canagliflozin [149,169,170]. Concerns over the impact of SGLT2-is on limb events were mainly triggered by the rise in toe and trans metatarsal amputations in the CANVAS program. The CANVAS program showed a statistically significant decrease in the risk of MACEs using canagliflozin but led to a twofold increase in the risk of amputations (HR 1.97; 95%CI: 1.41 to 2.75). Canagliflozin also had an increased risk of amputation and bone fracture compared to placebo. Patients who had a history of amputation or PAD had the greatest risk of amputation. It may be beneficial to use empagliflozin instead of canagliflozin in individuals with osteoporosis or previous amputation [158,171,172]. In a recent retrospective cohort study, new users of SGLT2-i had a significantly higher risk of amputation compared with metformin, sulfonylureas, and thiazolidinediones but not compared with new users of GLP-1 agonists or DPP-4 [169].

In two studies that compared SGLT2-i to GLP1-1 agonists, SGLT2-i were found to increase the risk of lower-limb amputation. Although GLP1-RA liraglutide is less likely to cause amputation than placebo [159], recent clinical data and large-scale real-world studies indicate that the link between amputation risk and canagliflozin use is less than previously described. The overall analyses have not indicated a significant increase in the risk of amputations associated with SGLT2-i. In 2020, the Food and Drug Administration removed the boxed warning about the risk of amputations for canagliflozin [155]. Although the Food and Drug Administration has eliminated the boxed warning linking canagliflozin to the risk of leg and foot amputation, the risk remains. The potential risks of SGLT2-i must be balanced with their potential benefits, including improved glycemic control and reduced rate of MACEs as demonstrated for empagliflozin in the EMPA-REG OUTCOME trial, for canagliflozin in the CANVAS program, and dapagliflozin in the DECLARE-TIMI trial. Therefore, health professionals should continue to recognize the importance of preventive foot care. In patients with advanced ATS and impaired peripheral circulation, canagliflozin should be avoided as it is still recommended [149,173,174].

## 12. Clinical Evidence of the Use of SGLT2-i in Diabetic Foot: State of the Art and Conclusions

**Figure 3:**
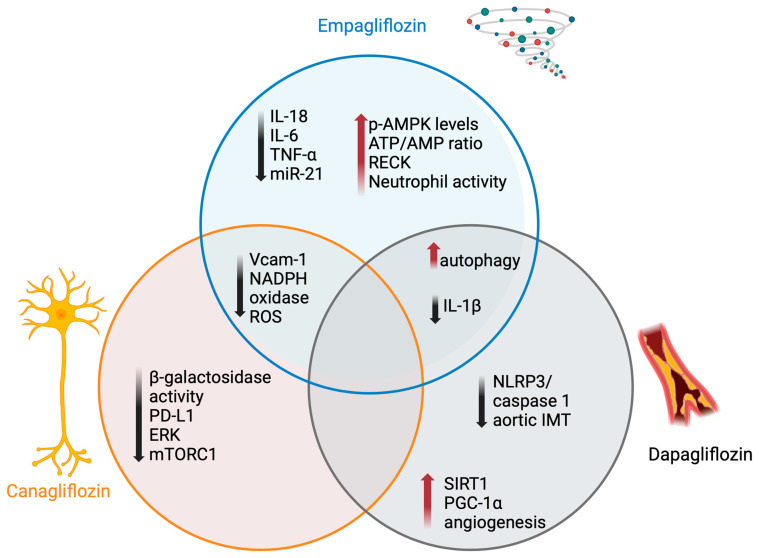
Main molecular mechanisms and effects of empagliflozin, canagliflozin, and dapagliflozin on immunomodulation, neuropathy, and angiopathy in diabetic patients. (Created with BioRender.com).

DFU is the first complication of lower-limb involvement in diabetes, the leading cause of death in diabetic patients, with a 10-year mortality of 71% [175], and the main risk factor for limb amputation [3]. For this reason, many efforts have been made to control the risk factors increasing the incidence of ulcer formation and enhance the therapeutic strategies leading to ulcer healing, even if the rates range from 48% to 86% at 52 weeks, with a higher impact on the disability and mortality in diabetic patients [176,177]. Based on the above data, we reported on the immunomodulatory and protective role of vascular and nerve function (summarized in Figure 3), and this review focuses on research data on the possible effect of SGLT2-i in preventing diabetic foot and its complications. This need comes from the evidence of the contrasting results from the CANVAS [144] and the CREDENCE studies [178] on the incidence of lower-limb amputation during the canagliflozin treatment and the boxed warning of the Food and Drug Administration to avoid the use of this medication in DFC. Several efforts were made to collect data proving the safety of SGLT2-i. This review aimed to find data in favor of or against the early use of SGLT2-i in DF. Although the large body of evidence demonstrates a favorable impact of SGLT2-i on the single mechanisms underlying the onset of DF, it is not possible to express an opinion on the potential clinical impact of this class of drugs in the prevention of DF due to the limited amount of available clinical data.

As shown below, several retrospective studies and meta-analyses confirmed the data from the CANVAS study regarding the higher risk of lower-limb complications. Lin et al. have performed a metanalysis whose aim was to analyze the impact of the use of SGLT2-i on lower-limb complications [149]. In the metanalysis, they included comprehensive RCTs of diabetic patients with lower-limb complications (PAD, DFU, or amputation) and a group of controls treated with placebo or other medications to compare the results. At the end of the enrollment, 39 studies were selected. The metanalysis showed that the use of SGLT2-i was associated with an increase in the incidence of PAD and amputation, while no association was found with diabetic foot incidence. However, the increased risk of PAD and amputation was found only in the subgroup treated with canagliflozin but not in other subtypes of SGLT2-i, and this was more evident in long-lasting treatment (over 52 weeks). Furthermore, the meta-regression analysis showed that the incidence of all three lower-limb complications was significantly associated with more significant weight reduction, lower diastolic baseline blood pressure, and a more substantial reduction in systolic and diastolic pressure in the SGLT2-i group. Given the effect on the hemodynamic balance, the check of weight and blood pressure variations might fall under the assessment of the prevention measures of diabetic foot complications in patients under SGLT2-i treatment. Weight loss from SGLT2-i treatment comes from enhanced diuresis and the glycosuric effect in the later stages [179]. However, the main determinant in the increased risk of amputation is the insufficient distal perfusion caused by depletion volume rather than weight loss. In support of this, the use of thiazide diuretics was also associated with an increased risk of amputation in T2DM [151]. On the other hand, Cai et al. demonstrated that, at the same weight reduction in both the GLP-1RA- and SGLT2-i-treated groups, only the latter presented an increase in amputation rates [180]. This result is confirmed in a real-world study [181] and might be explained by the lack of diuretic effect of the GLP-1 agonist. Weight reduction and decrease in blood pressure cause a depletion in vascular volume. Hence, they are responsible for the altered distal perfusion and the higher incidence of PAD and amputation. However, markers of volume depletion, such as hemoglobin and hematocrit, are not associated with a higher risk of peripheral complication, even if it could be due to the lack of significant available data.

Among SGLT2-i, canagliflozin is the only one associated with a higher amputation incidence. It might be explained by the higher weight loss and blood pressure reduction induced by canagliflozin compared with the other subtypes, possibly also due to the dual action on the SGLT1 [182], even if sotagliflozin, another SGLT1-SGLT2-i, did not show this association [183]. Another meta-analysis of 42 RCTs comparing the use of SGLT2-i (canagliflozin 100–300 mg/day, dapagliflozin 10 mg/day, empagliflozin 10–25 mg/day, ertugliflozin 5–15 mg/day, ipragliflozin 25–50 mg/day, luseogliflozin 2.5–5 mg/day, and tofogliflozin 20 mg/day) vs. placebo has shown a higher incidence in the onset of PAD, lower-limb fractures, or symmetric polyneuropathy even if it was without significative differences; furthermore, a significant increase in local ulcers, overall infections, and amputations was observed in the intentional group compared with the controls, underlying the need for physicians to be careful in the case of starting or continuing SGLT2-i in patients at higher risk of lower-limb complications [184]. A retrospective cohort study recruited 287,091 patients randomized into two groups, one treated with GLP1RA and the other with SGLT2i. The study’s primary endpoint was the MALE (major adverse limb events: composite of newly diagnosed critical limb ischemia, percutaneous transluminal angioplasty or peripheral bypass for peripheral artery disease, and nontraumatic amputation). At the end of the study, patients treated with GLP1RA had less incidence of major adverse limb events within the first two years from initiation, compared with the SGLT2-i group, in particular, in those affected with neuropathy [185]. So, these studies support the best medical choice might be avoiding the use of SGLT2-i in patients with diabetic foot, preferring other ADD. However, on the other hand, recent studies showed a sure profile of SGLT2-i without finding any association between the use of these medications and a higher risk of DFC, as demonstrated by the following.

In 2023, D’Andrea et al. [186] performed a comparative effectiveness and safety research study to evaluate the efficacy of SGLT2-i and safety compared with DPP-4i. A total of 87,274 patients were enrolled in the study and subdivided into two subgroups, based on the treatment with an SGLT2-i (canagliflozin, dapagliflozin, empagliflozin, or ertugliflozin) or a DPP-4i (alogliptin, saxagliptin, linagliptin, or sitagliptin). Among the safety items, the rates of risks of hypovolemia, nonvertebral fractures, falls, and lower-limb amputations were caught in and appeared to be similar in the two groups. Hence, SGLT2-i were shown to be as safe as DPP-4i, not increasing the incidence of lower-limb complications. The EMPRISE study is a sequentially built new-user, parallel-group, active-comparator retrospective cohort study that confirmed the safety of the use of empagliflozin compared with DDP-4i, regarding which no differences were found in the incidence of lower-limb amputations or fractures [187]. A retrospective study [188] selected a cohort of more than 3 million patients with T2DM divided into five non-overlapping anti-diabetic therapy groups (patients treated with SGLT2-i in association or not with DPP4-I or GLP-1RA; patients treated with GLP-1RA and no previous exposure to other anti-diabetic drugs; patients treated with DPP4-I; other anti-diabetic drugs; no ADD) and concluded that the use of SGLT2-i was not associated with a higher risk of lower-limb complications and no difference was found between the SGLT2-i classes. Moreover, patients treated with SGLT2-i in association with GLP1-RA had a lower incidence of PAD, which is the leading risk factor for amputation.

Lastly, a very recent retrospective study analyzing data from the Taiwan National Health Insurance Research Database (2004–2017), which recruited patients affected with T2DM and without previous MACEs and recently diagnosed DFC, confirmed decreased rates of lower-limb amputation in these patients, compared with DPP-4i therapy. This result suggests that SGLT2-i might be safe and effective in reducing the risk of amputation in patients affected with recent DF complications but with a low cardiovascular risk [189].

Therefore, univocal data on the safety of the SGLT2-i in diabetic foot have not been available until now. Considering the proven effectiveness of this class of anti-diabetic drugs on cardiovascular events and kidney protection, the acknowledgment of the impact of these medications on the worsening of lower-limb complications is essential for selecting the population to treat, avoiding the adverse effects without lowering the beneficial impact. Moreover, considering that some of the DF complications, such as osteomyelitis and DFU, are rapidly evolving and dangerous situations for diabetic patients, the knowledge of the causative mechanisms based on the correlation between SGLT2-i and their potential risk helps prevent irreversible damage. Hence, there is a need to obtain reliable results for the efficacy and safe use of these drugs with other studies. Despite the evidence demonstrating the favorable effects of SGLT2-i on the main complications of T2DM underlying the development of diabetic foot, no prospective longitudinal study has evaluated the reduction in the risk of developing DF in patients treated with SGLT2-i compared to other treatments. At the moment, most of the data available to us concern the safety profile of this class of drugs and the focus is on the risk of amputation. In light of the evidence reported above, it cannot be excluded that SGLT2-i and canagliflozin, in particular, may have a different effect on macro- and microvascular complications depending on the stage of progression of the vascular disease in the diabetic patient. If the increased risk of amputations in PAD patients treated with canagliflozin was confirmed, considering the promising data on the protective role of SGLT2-i on neurological, vascular, and immunomodulatory complications, it could be hypothesized that the dual detrimental and beneficial effect could depend on the duration of the disease and on the moment of the natural history in which the drug is inserted. In this sense, an early introduction before the onset of neuropathy and vasculopathy could enhance the protective role of SGLT2-i, and vice versa, a late introduction in more advanced patients could highlight the detrimental effects perhaps linked to hypovolemic alterations. Interestingly, in the CANVAS study, the highest absolute risk of amputation occurred among patients who had a history of amputation or peripheral vascular disease, but the relative risk of amputation with canagliflozin as compared with placebo was similar across these subgroups.

One of the main confounders that may affect the conflicting results is that the available data are collected from retrospective studies carried out to prove the efficacy of SGLT2-i on cardiovascular risk, while the effect on lower-limb complications is obtained as secondary data. Prospective and longitudinal observational studies with the primary aim of evaluating the incidence of DF complications during SGLT2-i treatment still need to be included to prove the results of the above research. These types of study may help to better evaluate the impact of SGLT2-i treatment on lower-limb complications, stratifying and selecting the population of study over strict criteria of inclusion, observing the patients throughout the treatment, and monitoring the onset of new events at the same time.

Moreover, all the studies taken into the exam have evaluated patients already affected with DFC, while no study has been provided to assess the possible protective role of SGLT2-i in preventing lower-limb complications if started at the onset of diabetes. Most of the studies and clinical trials evaluate the efficacy and safety of SGLT2-i by enrolling patients affected by long-term diabetes with high cardiovascular risk, while no studies have been conducted on the impact of SGLT2-i on recent diagnoses and low cardiovascular risk.

## 13. Conclusions

SGLT2-i have been shown to slow ATS progression by reducing inflammation and oxidative stress, independently of glucose control. They reduce proinflammatory cytokines, improve endothelial function, suppress oxidative stress, and inhibit key inflammatory pathways like the NLRP3 inflammasome. Additionally, these drugs promote autophagy, improve mitochondrial function, and stabilize atherosclerotic plaques, offering potential cardiovascular protection in diabetes. Recent studies also indicate that SGLT2-i may benefit diabetic neuropathy by targeting inflammation and oxidative stress pathways, independent of glucose regulation. For the abovementioned reasons, they are widely recognized for their cardioprotective and nephroprotective benefits in patients with T2DM. Emerging research also suggests that these drugs may have neuroprotective effects, particularly in diabetic neuropathy, where they improve nerve conduction and alleviate clinical symptoms. However, safety concerns persist, with certain studies showing a threefold increased risk of genital infections. In contrast, evidence linking SGLT2-i to UTIs is mixed, with some studies showing no increased risk compared to placebo. Despite these risks, SGLT2-i show promise in addressing other complications of diabetes, such as improving PAD outcomes and aiding in wound healing. Their anti-inflammatory and immunomodulatory effects, including influencing macrophage and T cell activity, may further contribute to their broad therapeutic potential, though more research is needed to fully assess the long-term effects and optimize their use. In conclusion, regarding the state of the art, there is good evidence for the single immunomodulatory, neuroprotective, and beneficial vascular effects of SGLT2-i. Still, there is no clinical evidence on diabetic foot due to the lack of longitudinal and prospective studies proving the effect of these drugs without confounders. The risk of limb amputation, not confirmed and limited to canagliflozin, has potentially discouraged the design of specific studies targeting diabetic foot. In light of this evidence, specific trials that consider the use of SGLT2-i in patients with PAD and prospective studies aimed at evaluating the potential protective effect of these drugs against the onset of diabetic foot are necessary.

## Data Availability

Not applicable.

## Abbreviations

ABI: ankle–brachial index; AMPK: adenosine 50-monophosphate-activated protein kinase; ATP: adenosine triphosphate; ATS: atherosclerosis; BP: blood pressure; DF: diabetic foot; DFS: diabetic foot syndrome; DFU: diabetic foot ulcers; DPN: diabetic peripheral neuropathy; DPP4i: dipeptidyl peptidase-4 inhibitors; ERK: extracellular signal-regulated kinases; ICAM-1: intercellular adhesion molecule 1; MACE: major adverse cardiovascular events; MCP-1: monocyte chemoattractant protein 1; MAPK: suppressed mitogen-activated protein kinases; MRSA: methicillin-resistant S. aureus; NADPH: nicotinamide adenine dinucleotide phosphate; NOX: NADPH oxidase; NHE3: sodium–hydrogen exchanger 3; NO: nitric oxide; PAD: peripheral artery disease; PCT: proximal convoluted tubule; PECAM-1: platelet endothelial cell adhesion molecule-1; PKC: protein kinase C; PWV: pulse wave velocity; RANKL: receptor of nuclear factor (NF)-ĸB ligand; ROS: reactive oxygen species; SGLT2-i: sodium–glucose cotransporter 2 inhibitors; T2DM: type 2 diabetes mellitus; TmG: transport maximum renal glucose reabsorption; UTI: urinary tract infection; VCAM: vascular cell adhesion molecule.
